# Gender, Social Ties, and Older Adult Alcohol Use

**DOI:** 10.1093/geroni/igaf122.3452

**Published:** 2025-12-31

**Authors:** Eric Vogelsang, Zackary Zanotelli

**Affiliations:** California State University, San Bernardino, San Bernardino, California, United States; California State University, San Bernardino, San Bernardino, California, United States

## Abstract

There is little empirical research focusing on how community, family connections, and gender may influence the risk of older adult alcohol use. This is important, given growing concerns over (a) the long-term effects of even moderate alcohol use worldwide, (b) growing alcohol consumption among women, and (c) health impacts of social isolation. Using The Irish Longitudinal Study on Ageing (18,805 observations from 7,705 respondents), we examine how “social ties” (family characteristics and social participation) predict three measures of alcohol use and abuse among adults aged 50-plus. We also test to what extent alcohol use and its correlates may differ by gender. Our results indicate that having three or more close friends (when compared to having no close friends) is associated with regular alcohol consumption, and (for men) greater odds of possible alcohol abuse. We also find that, for women, being a member of a social organization is associated with greater odds of drinking, when compared to those who are not. Unlike similar prior studies, religious participation (which was more common among women) was negatively associated with all three types of alcohol consumption. Last, and contrary to expectations, greater education was positively associated with all three measures of alcohol use and abuse. In total, these findings suggest one potential drawback to close social ties in older ages. They also suggest ways in which a country’s history and gendered social norms may influence health behavior in later life.

